# Implications of quantitative susceptibility mapping at 7 Tesla MRI for microbleeds detection in cerebral small vessel disease

**DOI:** 10.3389/fneur.2023.1112312

**Published:** 2023-03-15

**Authors:** Valentina Perosa, Johanna Rotta, Renat Yakupov, Hugo J. Kuijf, Frank Schreiber, Jan T. Oltmer, Hendrik Mattern, Hans-Jochen Heinze, Emrah Düzel, Stefanie Schreiber

**Affiliations:** ^1^J. Philip Kistler Stroke Research Center, Massachusetts General Hospital, Boston, MA, United States; ^2^Department of Neurology, Otto-von-Guericke University, Magdeburg, Germany; ^3^Institute of Cognitive Neurology and Dementia Research (IKND), Magdeburg, Germany; ^4^German Center for Neurodegenerative Diseases (DZNE), Magdeburg, Germany; ^5^Image Sciences Institute, University Medical Center Utrecht, Utrecht, Netherlands; ^6^Athinoula A. Martinos Center, Massachusetts General Hospital, Department of Radiology, Boston, MA, United States; ^7^Institute of Physics, Otto-von-Guericke University, Magdeburg, Germany; ^8^Center for Behavioral Brain Sciences, Magdeburg, Germany; ^9^Institute of Cognitive Neuroscience, University College London, London, United Kingdom

**Keywords:** 7 Tesla MRI, cerebral small vessel disease (CSVD), cerebral amyloid angiopathy (CAA), microbleeds, quantitative susceptibility mapping (QSM), hypertensive arteriopathy (HA)

## Abstract

**Background:**

Cerebral microbleeds (MBs) are a hallmark of cerebral small vessel disease (CSVD) and can be found on T2^*^-weighted sequences on MRI. Quantitative susceptibility mapping (QSM) is a postprocessing method that also enables MBs identification and furthermore allows to differentiate them from calcifications.

**Aims:**

We explored the implications of using QSM at submillimeter resolution for MBs detection in CSVD.

**Methods:**

Both 3 and 7 Tesla (T) MRI were performed in elderly participants without MBs and patients with CSVD. MBs were quantified on T2^*^-weighted imaging and QSM. Differences in the number of MBs were assessed, and subjects were classified in CSVD subgroups or controls both on 3T T2^*^-weighted imaging and 7T QSM.

**Results:**

48 participants [mean age (SD) 70.9 (8.8) years, 48% females] were included: 31 were healthy controls, 6 probable cerebral amyloid angiopathy (CAA), 9 mixed CSVD, and 2 were hypertensive arteriopathy [HA] patients. After accounting for the higher number of MBs detected at 7T QSM (Median = Mdn; Mdn_7T−QSM_ = 2.5; Mdn_3T−T2_ = 0; *z* = 4.90; *p* < 0.001) and false positive MBs (6.1% calcifications), most healthy controls (80.6%) demonstrated at least one MB and more MBs were discovered in the CSVD group.

**Conclusions:**

Our observations suggest that QSM at submillimeter resolution improves the detection of MBs in the elderly human brain. A higher prevalence of MBs than so far known in healthy elderly was revealed.

## Introduction

Pathological changes proper of cerebral small vessel disease (CSVD) lead to leaks and rupture of the vessel's wall, sometimes resulting in the accumulation of intact erythrocytes or hemosiderin (1, 2). These acute, subacute or chronic small focal lesions are called cerebral microbleeds (MBs) and are among the most representative hallmarks of CSVD (3, 4). As such, they correlate with the pathological burden of the disease (5, 6), are predictive of the risk of intracerebral hemorrhage (7, 8) (ICH), which is the most acute and devastating outcome of sporadic CSVD, and show an inconsistent association with cognitive impairment (9, 10). The distribution of cerebral MBs in the human brain also creates patterns that allow the distinction between the two most common forms of sporadic CSVD: Hypertensive arteriopathy (HA), which is associated with hypertension and manifests especially in the perforating vessels of the basal ganglia (11), and cerebral amyloid angiopathy (CAA), which mainly affects the leptomeningeal and cortical small arteries and is characterized by the accumulation of amyloid β (Aβ) (12). Consequently, MBs are typically to be found in the deep brain regions in HA, whereas they are strictly lobar (and mostly cortical) in CAA (11). Mixed patterns, which likely express the co-occurrence of both vessel pathologies, also exist (13). Moreover, MBs are not rare in Alzheimer's disease (~25%) (14, 15) and recent developments also suggest that MBs and the higher risk for brain hemorrhage that they are linked to, might encourage caution for the recently approved anti-Aβ Alzheimer's disease therapies (16). MBs are present also in healthy elderly adults, increasing from approximately 17% in the sixth to up to 38% in the eighth decade of life (17).

MBs are routinely detected on MRI using T2^*^-weighted (T2^*^-w) sequences and susceptibility weighted imaging (SWI). Quantitative susceptibility mapping (QSM) (18), a relatively novel post processing method, offers advantages, such as the absence of the blooming effect proper of T2^*^-w sequences and SWI (19) and the possibility to discriminate between diamagnetic and paramagnetic substances (20) (e.g., between calcium—as found in calcifications—and hemosiderin deposits—as found in MBs). In this study, we hypothesize that MBs detection would benefit from (i) greater magnetic field strength (3T T2^*^-w vs. 7T T2^*^-w imaging), as previously shown (21), and (ii) the use of QSM (7T T2^*^-w vs. 7T QSM). Additionally, in the same cohort of patients with CSVD and healthy elderly participants, we explore the implications of 7T QSM for the neuroimaging-based classification of patients as CSVD and/or controls.

## Methods

### Participants

Patients with CSVD were selected within a longitudinal 3T MRI study on the pathophysiology of CSVD conducted at the University Clinic of Magdeburg and German Center for Neurodegenerative Disease (DZNE), Magdeburg. Presence of hemorrhagic CSVD markers, i.e., MBs, ICH and/or cortical superficial siderosis (cSS) were the screening criteria for inclusion in this study. The lesions were individuated on iron-sensitive MRI sequences [gradient recalled echo (GRE) T2^*^-weighted or susceptibility weighted imaging (SWI)] of a clinical 1.5T MRI conducted for diagnostic work-up. Reasons for the diagnostic MRI included epileptic seizures, gait disturbances, cognitive impairment, headache, and transient ischemic attack.

Controls were recruited from a pool of cognitively normal community-dwelling elderly individuals of the DZNE, Magdeburg, who previously participated in one or more aging studies and, within this frame, underwent a 3T MRI scan that involved a susceptibility sensitive MRI, on which no hemorrhagic markers (MBs, ICH, cSS) were found (22).

After this initial recruitment all participants of this study underwent a clinical neurological visit, during which genetic neurological disease, history of psychiatric disease, alcohol or drug abuse, and cerebrovascular malformations were excluded. Cardiovascular risk factors were also gathered for all participants. Arterial hypertension was defined as >130/80 mmHg. Diabetes mellitus was diagnosed in case fasting plasma glucose level was >7.0 mmol/L or > 11.1 mmol/L 2 h after glucose tolerance test. Hyperlipidemia was recorded when there were abnormal blood levels of low-density lipoprotein cholesterol (>2.6 mmol/L) and/or triglycerides (>1.7 mmol/L).

Furthermore, both a 3T and a 7T MRI scan were conducted. Contraindications for scanning at 7T were considered and represented a further exclusion criterion from our study, according to the recommendations of the German Ultrahigh Field Imaging network (www.mr-gufi.de).

### 3T MRI

Participants were subject to a 3T scan prior to 7T MRI [median interval (range) in months: 2.5 (1–25)], which was performed in a Siemens Verio scanner with a Siemens 32-channel array coil. The protocol included a T2^*^-w 3D GRE pulse sequence (voxel size: 1 x 1 x 2 mm^3^, echo time: 20 ms, repetition time: 28 ms, flip angle: 17°, receiver bandwidth (RBW) 100 Hz/px, GRAPPA with factor 2, 24 reference lines were enabled, scanning time 5.23 min), which served for the identification of cerebral MBs, cSS, and ICH. A T2-weighted fluid-attenuated inversion recovery (FLAIR) sequence was included to determine white matter hyperintensity (WMH) patterns (see below, scanning time 7.02 min). (23) A T2-weighted turbo spin echo sequence was used to establish the burden of enlarged perivascular spaces (EPVS) in the centrum semiovale (CSO) (scanning time 5.40 minutes) (24). Total scanning time amounted to 45 min, because the protocol included further sequences not implicated in this study.

### 7T MRI

7T MRI was conducted at a Siemens Healthineers, scanner equipped with a 32-channel head-coil (Nova Medical). A T2^*^-w 3D GRE sequence was acquired (voxel size: 0.35 x 0.35 x 1.5 mm3, echo time: 9 ms, repetition time: 18 ms, flip angle: 10°, RBW 100 Hz/px; 3D matrix dimensions 200 x 169 x 132. GRAPPA enabled with an acceleration factor of 2 and 32 reference lines, scanning time 5.48 min). A 3D-MPRAGE was also acquired for anatomical reference (scanning time 5.23 min). The protocol included more sequences that were not used in our study. Total scanning time was 50 min, because the protocol included further sequences not adopted in this study.

### QSM reconstruction from 7T T2^*^-w imaging

As previously explained in another study by our group (25), the T2^*^-w magnitude and phase data underwent reconstruction steps, in order to produce QSM maps. Multiple-channel complex image data were combined using an adaptive algorithm (26) followed by automatic reference channel selection. Unwrapping of the combined phase data was achieved using a continuous Laplacian approach (27). Adopting FSL's BET routing (threshold 0.1), a brain mask was calculated from the magnitude image and applied to the phase image. The background field was consequently removed in two steps: Laplacian boundary value (LBV) (28) with two-layer region of interest (ROI)-peeling, followed by variable mean spherical value (vSMV) (29) with r0 = 40 mm and step size/final kernel radius of 1 mm. Finally, a Multi-Scale Dipole Inversion (MSDI) (30) was carried out in order to reconstruct quantitative susceptibility maps from the local field maps obtained in the previous step.

### Assessment of neuroimaging markers

**Figure 1:**
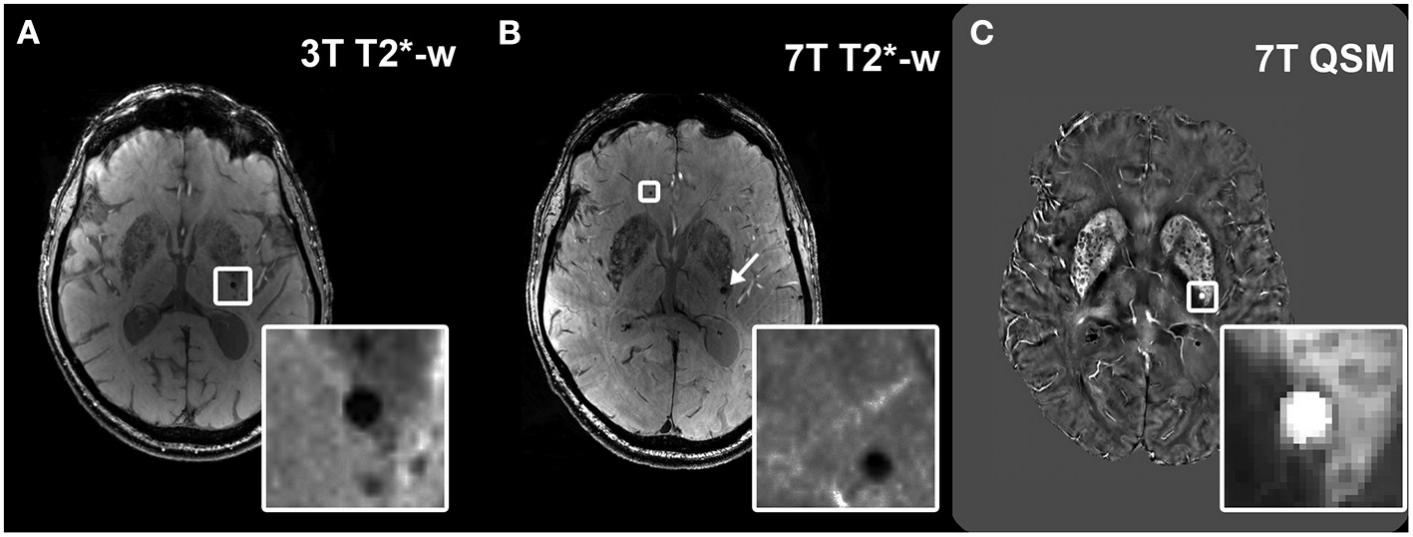
Visualization of microbleeds on different sequences. Example of a microbleed (MB) visualized as a hypointense round structure in the deep brain region at 3T T2*-w imaging **(A)**; visualization of the same brain region in the same subject at 7T T2*-w imaging, which shows the same MB (arrow) and an additional MB that had remained undetected at 3T (inset) **(B)**. Hyperintense appearance of the deep MB (inset) in the same subject on 7T QSM **(C)**.

In this study, neuroimaging markers of CSVD were assessed. **Microbleeds (MBs)** were defined as small, round lesions of 2–5 mm in diameter that appear hypointense on T2^*^-w imaging and hyperintense on QSM (Figure 1). Because of its quantitative nature, QSM allows to distinguish between the susceptibility provoked by a diamagnetic substance, and that provoked by a paramagnetic substance. Therefore, **calcifications**, which resemble MBs on T2^*^-w magnitude images, can be distinguished from hemosiderin deposits. In our study, calcifications were defined as small (2–5 mm) hypointense round lesions on 7T QSM. Each calcification identified on 7T QSM was also compared to the respective 7T T2^*^-w magnitude image, where it also appears hypointense, in order to ensure that the same lesion had been identified there as a MB. The localization of the MBs was recorded according to the Microbleed Anatomical Rating Scale (MARS) as lobar (frontal, parietal, temporal, occipital, insular), deep (deep and periventricular white matter, basal ganglia, thalamus, internal capsule, external capsule and corpus callosum) or infratentorial (brainstem, cerebellum).

Additionally, the total number of MBs was recorded. **Cortical superficial siderosis (cSS)** and **intracerebral hemorrhage (ICH)** were identified on the 3T T2^*^-w magnitude images sequence. Furthermore, **enlarged perivascular spaces (EPVS)**, which are thin hyperintense stripes or ovals up to 3 mm, and **white matter hyperintensities (WMH)** were also assessed on T2-weighted and FLAIR MRI respectively, because they are part of the most recent version of the Boston criteria for the diagnosis of CAA (31). In fact, severe EPVS in the CSO (> 20 in one hemisphere and one axial slide) and/or a multi-spot WMH pattern [>10 small circular or ovoid T2/FLAIR-hyperintense lesions in the bilateral subcortical white matter (32)] can determine possible or probable CAA, assuming the other diagnostic criteria are met (31).

Participants were classified in four CSVD subgroups according to the presence and distribution of hemorrhagic markers on 3T T2^*^-w imaging, of EPVS in the CSO, and of multispot WMH for the CAA sub-group. All CSVD participants had at least one hemorrhagic marker. The first sub-group was that of possible CAA, which according to the Boston criteria 2.0 (31), implies the presence of one strictly lobar MB, or one lobar ICH, or cSS, or of severe burden of CSO-EPVS and/or multi-spot WMH, accompanied by a clinical manifestation related to CAA (cognitive impairment, spontaneous ICH or transient focal neurological event). Probable CAA is on the other hand defined by the presence of >1 strictly lobar hemorrhagic marker (MBs, cSS or ICH) or one lobar MBs and either severe EPVS in the CSO or a multispot WMH pattern. For this diagnosis, deep MBs/ICH are not allowed. A further group was that of hypertensive arteriopathy (HA), which was defined as presence of strictly deep MBs (13). Finally, the mixed CSVD group involved the presence of both mixed lobar and deep MBs (13, 33).

Healthy controls were considered as such when they had no hemorrhagic markers (MBs, ICH, cSS), no severe EPVS in both the BG and CSO, no multi-spot WMH and were recruited from an already existing pool of cognitively normal community-dwelling elderly.

The baseline classification of the participants was carried out on 3T T2^*^-w images. The hypothetical reclassification of the cohort, as a consequence of the detection of further MBs and discrimination from calcifications, was based on 7T QSM. The 7T T2^*^-w sequence was not used for the purposes of classification in subgroups.

### Data availability

De-identified data are available upon reasonable request subject to a material transfer agreement.

### Statistics

Shapiro-Wilk-Tests were used to determine the distribution of the data, which proved to be non-normal in all cases. We therefore applied the Wilcoxon-Signed Rank test to evaluate differences between the number of MBs detected at 3T T2^*^-w vs. 7T T2^*^-w imaging, 7T T2^*^-w imaging vs. 7T QSM, and 3T T2^*^-w imaging vs. 7T QSM. Moreover, percentages of the distribution of the calcifications in different areas of the brain and with respect to the total number of MBs on 7T T2^*^-w were also calculated. Statistical analyses were two-tailed and conducted using IBM SPSS Statistics version 23.

## Results

**Figure 2:**
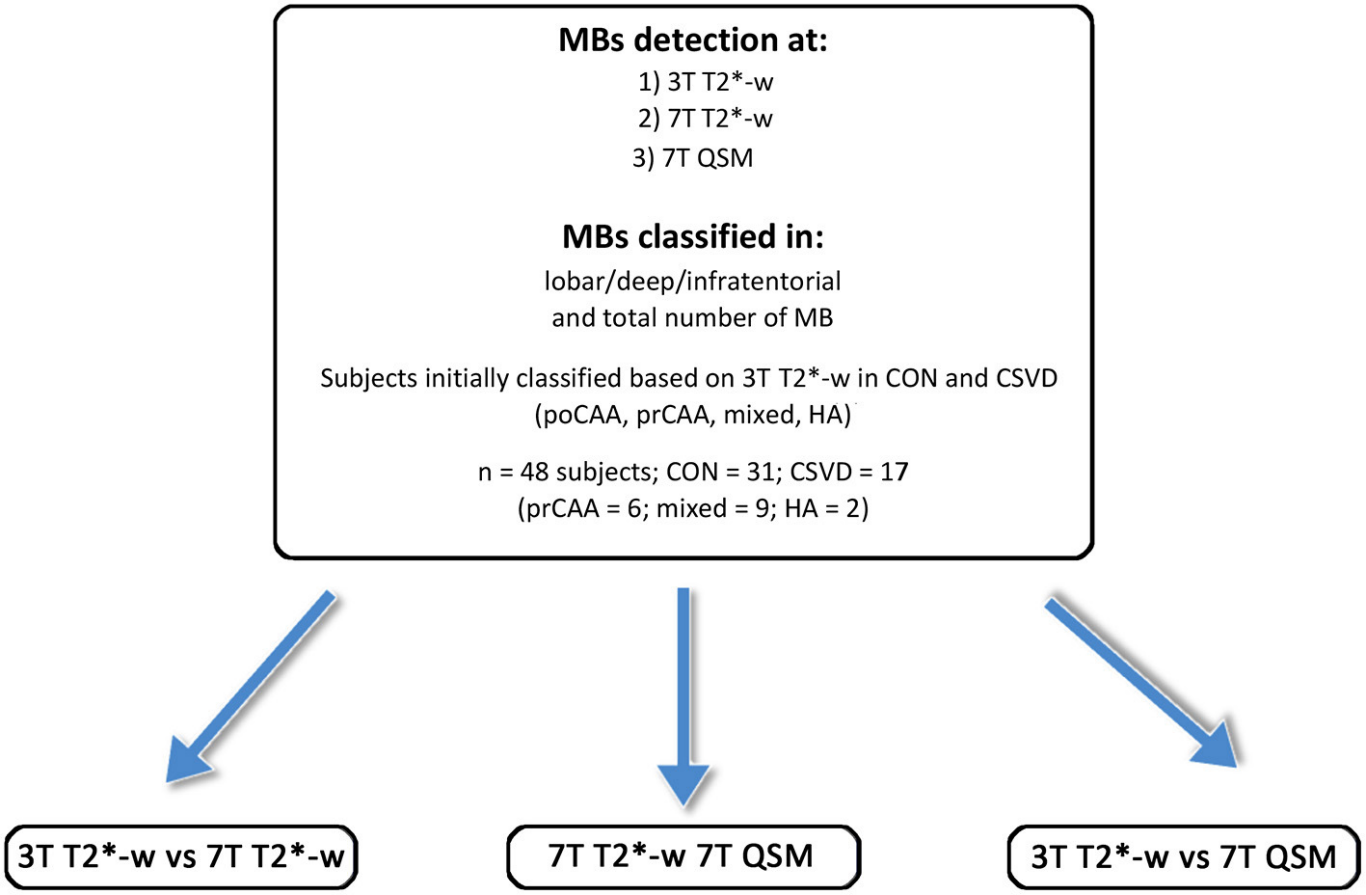
Overview of the study participants, the subgroups and the MRI scans performed. CAA, cerebral amyloid angiopathy; poCAA, possible CAA; prCAA, probable CAA; CON, controls; HA, hypertensive arteriopathy.

Based on 3T T2^*^-w imaging, a total of 48 participants [mean age (SD) 70.9 (8.8) years, 48% females] were included. 31 participants were classified as healthy controls [69.4 (9.9) years, 42% females], 6 as probable CAA, 9 as mixed CSVD, and 2 were HA patients (Table 1 and Figure 2).

**Table 1:** Characteristics of the groups.

	**Probable CAA (*n =* 6)**	**HA (*n =* 2)**	**Mixed MBs (*n =* 9)**	**Controls (*n =* 31)**	**Group differences**
Age years	72.3 (+/− 6.3)	71.1 (+/− 4.3)	71.5 (+/− 7.3)	69.4 (+/− 9.9)	H(3) = 0.54, *p =* 0.91
Female %	50	0	33	42	H(3) = 1.76, *p =* 0.623
BMI (kg/m^2^)	27.0 (+/− 2.6)	31.4 (+/− 1.8)	26.9 (+/− 3.7)	25.2 (+/− 2.4)	H(3) = 7.19, *p =* 0.066
Diabetes mellitus %	17	0	44	10	H(3) = 4.55, *p =* 0.21
Arterial hypertension %	100	100	100	53	H(3) = 9.75, *p =* 0.021 *post hoc* tests not significant
Hyperlipidemia %	50	100	67	39	H(3) = 3.75, *p =* 0.29

The table reports the characteristics of the study participants based on 3T T2^*^-w imaging, including demographics and vascular risk factors. Mean and SD are given. Kruskal-Wallis test is reported, assessing differences between the subgroups.

BMI, body mass index; CAA, cerebral amyloid angiopathy; HA, hypertensive angiopathy; MBs, microbleeds.

### Number of MBs detected on 7T is higher than on 3T T2^*^-weighted MRI

**Figure 3:**
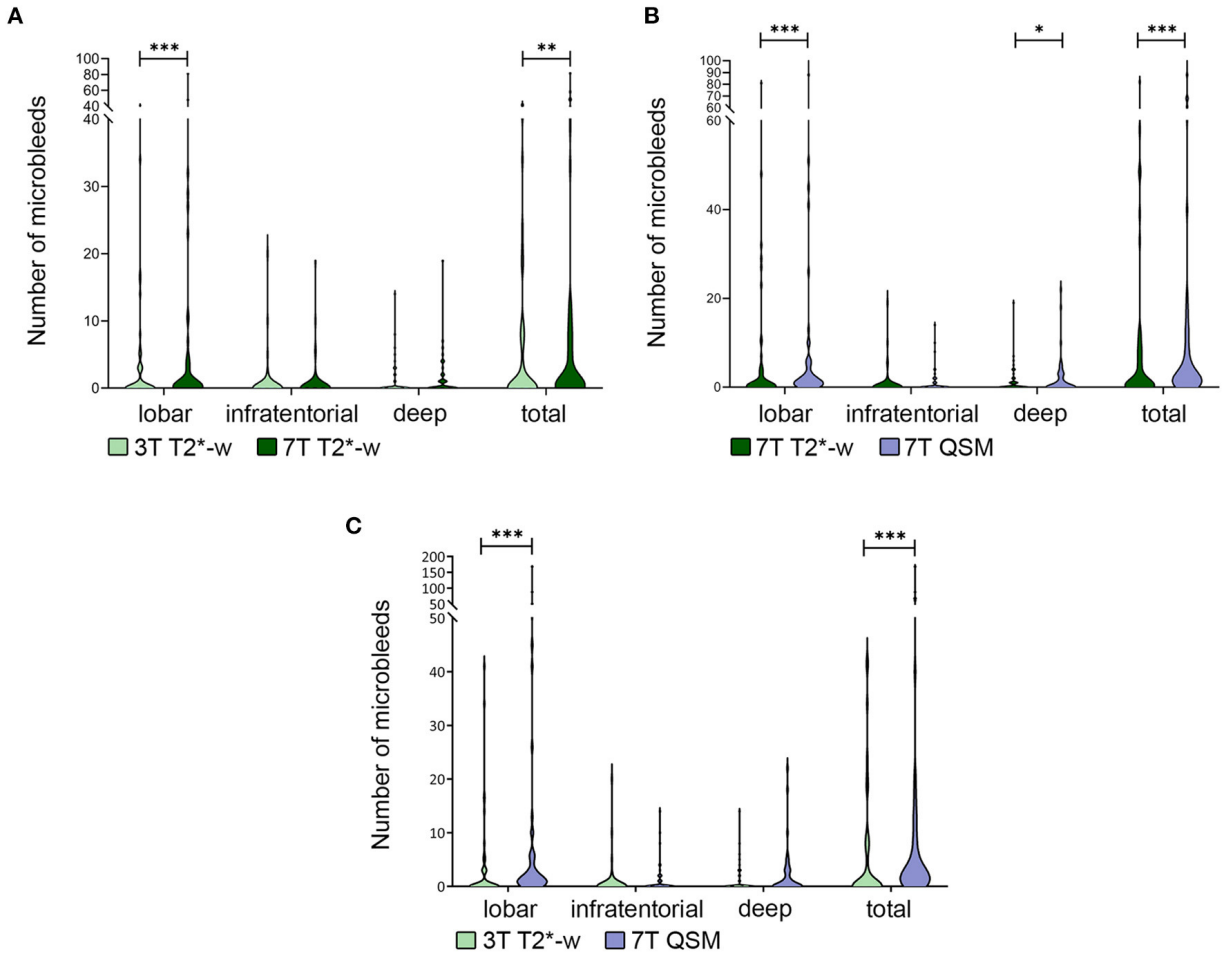
Differences in number of microbleeds. Legend: Violin plot showing the difference in total number of microbleeds (MBs) and in different brain localizations in 3T vs. 7T T2*-w imaging **(A)**, 7T T2*-w imaging vs. 7T QSM **(B)**, 3T T2*-w imaging vs. 7TQSM **(C)**. The difference in total number of detected MBs is always significant and driven by the difference in number of those in lobar localization. *p < 0.05; **p < 0.01; ***p < 0.001.

In our cohort the total number of MBs on 7T T2^*^-w magnitude images (Median = Mdn; Mdn = 1) was significantly higher than on 3T T2^*^-w imaging (Mdn = 0, *z* = 3.11, *r* = 0.32, *p* = 0.002). This difference was driven by lobar MBs (Mdn_3T−T2−w_ = 0, ${\text{Mdn}}_{7\text{T}T2^{*}-w}$ = 1, *z* = −3.58, *r* =−0.37, *p* < 0.001), while in deep (Mdn_3T − T2−w_ = 0, ${\text{Mdn}}_{7\text{T}T2^{*}-w}$ = 0, *z* = 0.66, *r* = 0.067, *p* = 0.508) and infratentorial (Mdn_3T−T2−w_ = 0, ${\text{Mdn}}_{7\text{T}T2^{*}-w}$ = 0, *z* = 1.80, *r* = 0.18, *p* = 0.072) regions the difference was not statistically significant (Figure 3A).

### Number of MBs detected on 7T QSM is higher than on 7T T2^*^-weighted MRI

**Figure 4:**
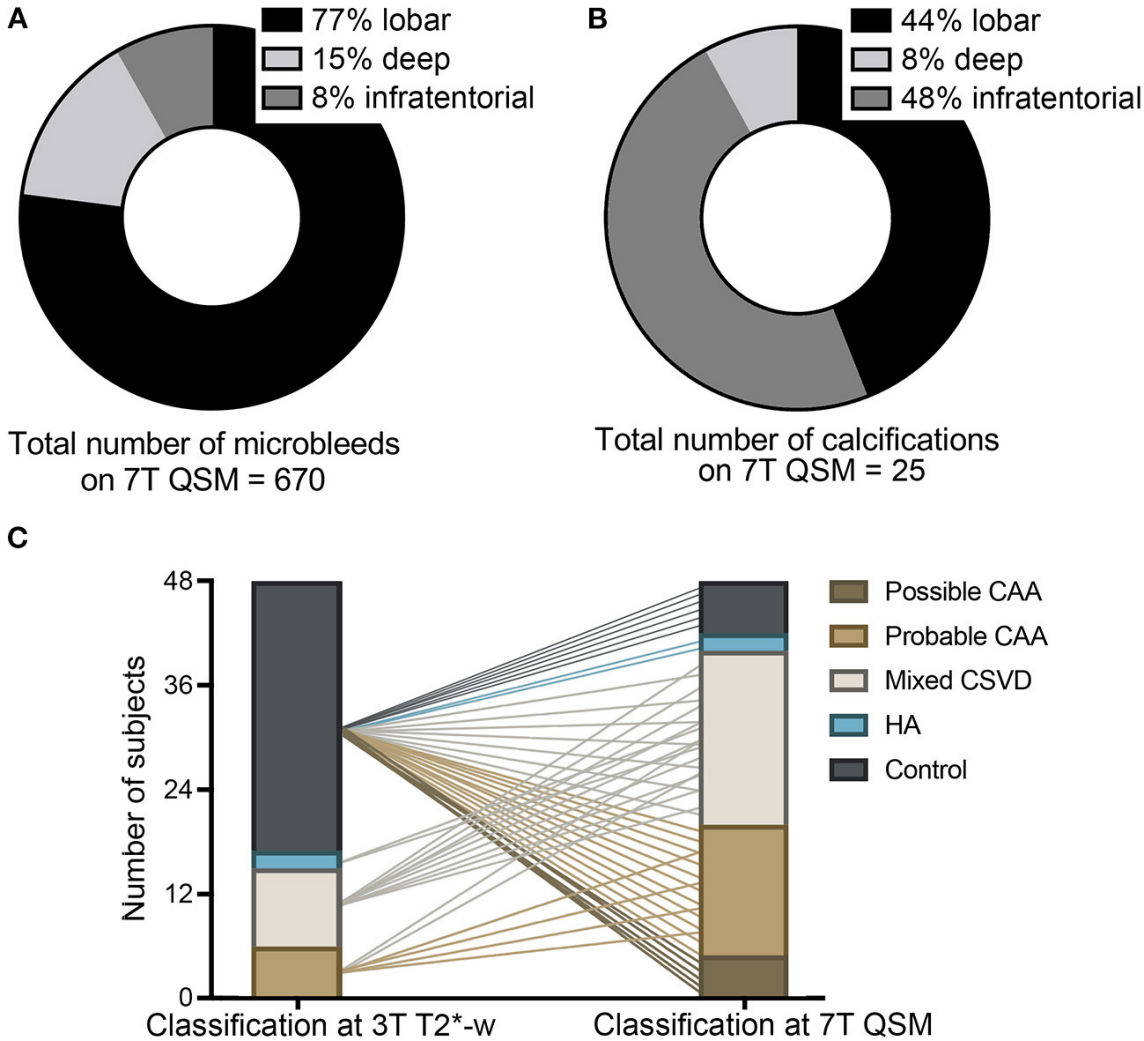
Implications of the use of 7T QSM. Legend: Pie charts showing the localization of the total amount of microbleeds (MBs) in our study cohort, as detected on QSM **(A)** and of calcifications **(B)**, as detected on 7T QSM. Graph **(C)** elucidating the classification of study participants in the different subgroups at 3T T2*-w imaging and how 7T QSM would impact the neuroimaging-based classification due to the increased number of MBs detected and due to the calcifications. Possible and probable cerebral amyloid angiopathy (CAA, light and dark brown), mixed cerebral small vessel disease (CSVD, white), hypertensive arteriopathy (HA, blue), healthy controls without CSVD (gray) **(C)**.

Furthermore, the use of 7T QSM overall allowed to recognize more MBs (Mdn_7T−QSM_ = 2.5) than the 7T T2^*^-weighted sequence (${\text{Mdn}}_{7\text{T}T2^{*}-w}$ = 1, *z* = 4.45, *r* = 0.46, *p* < 0.001). In this case the statistically significant difference concerned lobar MBs (${\text{Mdn}}_{7\text{T}T2^{*}-w}$ = 1, Mdn_7T−QSM_ = 1; *z* = 4.22, *r* = 0.43, *p* < 0.001), but was also relevant to deep brain regions (${\text{Mdn}}_{7\text{T}T2^{*}-w}$ = 0, Mdn_7T−QSM_ = 0; *z* = 1.98, *r* = 0.20, *p* = 0.047). In infratentorial brain regions, the observed differences in MBs count were not statistically significant between 7T T2^*^-w imaging and 7T QSM (Mdn_7T−QSM_ = 0; ${\text{Mdn}}_{7\text{T}T2^{*}-w}$ = 0, *z* = 1.48, *r* = 0.15, *p* = 0.139) (Figures 3B, 4A).

### Calcifications can be distinguished from MBs on 7T QSM

**Figure 5:**
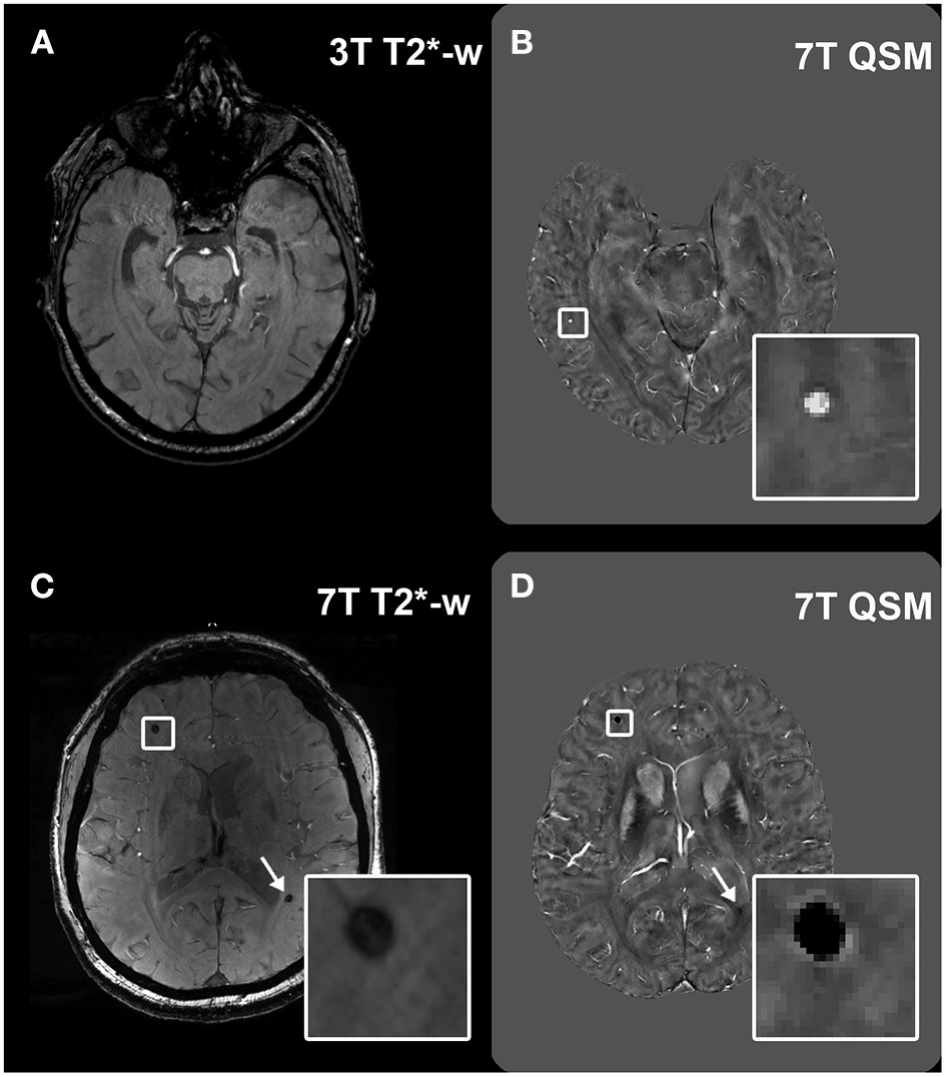
Visualization of lesions at 7T QSM. Legend: Example of a microbleed (MB) which was not visible at 3T T2*-w imaging in a healthy control **(A)**, but was then detected at 7T QSM [**(B)**, inset]. Calcifications can appear as hypointense round structures on T2*-w imaging, easily mimicking MBs [**(C)**, inset and arrow], however they can be differentiated from MBs in the QSM because of their hypointense appearance [**(D)**, inset and arrow].

On 7T QSM we could identify 25 calcifications (false-positive MBs) on a total number of 410 MBs detected at 7T T2^*^-w (6.1%) (Figure 5). They represented 3.6% (11/301) of the lobar MBs, 3% (2/66) of the deep and 27.9% (12/43) of the infratentorial MBs found on 7T T2^*^-w imaging. Calcifications were found in 29.8% (14/48) of all study participants, more specifically in 50% (3/6) of the probable CAA, 67% (6/9) of the mixed CSVD patients and 100% (2/2) of the HA patients, whereas this was the case in only 10% (3/31) of the controls. Between the subgroups the proportion of calcifications found was different: The average ratio of calcifications (number of calcifications on 7T QSM/number of MBs on 7T T2^*^-weighted) was 5% (8/161) in the probable CAA group, 4.5% (10/223) in the mixed CSVD cases, and as much as 60% (3/5) in the HA group, whereas these lesions represented 17% (4/23) of those found in the controls. Overall, calcifications were localized primarily in infratentorial (44%, 11/25) and lobar (48%, 12/25) regions, whereas only 8% (2/25) were found in deep brain regions (Figure 4B).

### 7T QSM leads to a shift in the neuroimaging-based classification of study participants

The total number of MBs was significantly higher on 7T QSM (Mdn = 2.5) than on 3T T2^*^-w imaging (Mdn = 0, *z* = 4.90, *r* = 0.50, *p* < 0.001). We counted more lobar MBs on 7T QSM (Mdn = 1) than on 3T T2^*^-w imaging (Mdn = 0, *z* = 5.01, *r* = 0.52, *p* < 0.001). The difference in MBs detection in deep (Mdn_3T−T2−w_ = 0, Mdn_7T−QSM_ = 0, *z* = 1.66, *r* = 0.17, *p* = 0.098) and infratentorial (Mdn_3T−T2−w_ = 0, Mdn_7T−QSM_ = 0, *z* = 1.80, *r* = 0.18, *p* = 0.072) brain regions was not significant (Figure 3C).

The baseline classification of the study participants in controls and CSVD subgroups, which was performed on 3T T2^*^-w imaging, demonstrated a shift when reassessed at 7T QSM, because of the increased number of MBs detected on this sequence and the discrimination between MBs and calcifications. As visualized in Figure 4C, 33% (2/6) of the probable CAA patients were reclassified to mixed CSVD cases because of the additional presence of deep MBs. The 2 HA patients turned out to be mixed CSVD cases due to the presence of lobar MBs. Most importantly, 80.6% (25/31) of healthy elderly controls showed at least one MB on 7T QSM, in this way fulfilling neuroimaging-criteria primarily for possible (20%, 5/25) and for probable CAA (44%, 11/25). The remaining participants were subclassified into mixed CSVD (20%, 5/25) and HA (8%, 2/25). This result suggests that most likely, the majority of healthy elderly presents cerebral MBs, which may reflect burden of CSVD pathology in advanced age.

## Discussion

In this study, we explored the implications of the use of QSM at high-field and high-resolution MRI for the detection of cerebral MBs. When compared to 7T T2^*^-w imaging, QSM at 7T allowed the detection of a higher number of MBs and the differentiation of MBs from calcifications. The combination of these factors revealed that the majority of healthy elderly participants showed one or more MBs, so fulfilling commonly applied neuroimaging-criteria for a CSVD subgroup.

As expected, a higher number of MBs was found at 7T QSM and at 7T T2^*^-w when compared to 3T T2^*^-w imaging, firstly, because higher field strength allows a better signal-to-noise ratio (SNR), and thus higher resolution and better visualization of smaller structures (21). This result is in line with previous works, which observed how MBs detection was superior on 7T compared to 1.5T (21) and 3T T2^*^- w imaging (34). In the present work, the 3T and the 7T scan were not conducted during the same session, so that it is possible that more MBs have developed in the participants during this time, especially in the participants who had a higher interval between MRI scans, which was up to 25 months. However, the median time gap between scans was only 2.5 months and according to a study on a memory clinic cohort, only 12% of the participants developed new MBs over 2 years (35). In our cohort, on the other hand, 60% of the participants showed more lesions on the 7T QSM compared to the 3T T2^*^-w imaging, making it very unlikely that this figure only depends on disease progression.

More MBs were found in the same subjects at 7T QSM when compared to 7T T2^*^-w imaging, indicating a higher sensitivity of the former. The feasibility of detection of MBs using QSM has been addressed before in the ArcAβ mouse model (36), and in human patients with Cushing's disease (37), multiple sclerosis (38), and traumatic brain injury (39). However, to the best of our knowledge, this is the first work that focused on the use of QSM at high-field MRI for MBs detection in patients with CSVD and healthy elderly controls. Subramaninan et al. argued that QSM could potentially better distinguish between MBs and aneurysms or other vessels' malformations when compared to T2^*^-weighted imaging. Similarly, a previous study from our group, which investigated an overlapping cohort, was able to distinguish MBs with a venous connection at 7T QSM (25).

One of the advantages of QSM, when compared to T2^*^-w imaging, is the ability to distinguish between hyperintense hemosiderin deposits and hypointense calcifications (40, 41). In our study cohort, 6% of the MBs at 7T T2^*^-w imaging proved in fact to be calcifications on 7T QSM.

Remarkably, 80.6% of healthy participants showed one or more MBs on QSM at 7T. In the Rotterdam-study, prevalence of MBs increased with age and was up to 38.3% in the eldest group of healthy controls (80–97 years old) (17). In our cohort this percentage was double as much, even though the mean age was lower (~70 years old) and the prevalence of vascular risk factors comparable to other cohorts. In further studies on community-based cohorts, this percentage was also considerably lower and was 8.8% in the Framingham Heart Study (42) and 5% in the Northern-Manhattan-Study (43). It must be kept in mind, that these numbers derive from population studies that adopted scanners with lower magnetic field than 7T. Nonetheless, this result points toward a possibly high prevalence of an at least mild degree of CSVD pathology in cognitively healthy elderly, who do not display further severe neuroimaging markers of CSVD. The presence of lobar and deep MBs discovered in the majority of healthy-elderly controls, could spur further and broader studies, involving the use of QSM, that better determine the clinical relevance of these observations. Considering that the current diagnostic criteria for CAA require the manifestation of a symptom and that they have been established on lower-field MRI (31), the importance of MBs in healthy elderly controls might not rest in the determination of a CSVD diagnosis. Instead, in this context MBs could become an alarm bell for undetected hypertension (42), high blood pressure variability, or heart disease (44, 45). Moreover, an investigation of the pathological correlates of the MBs found on 7T QSM would allow to determine their actual cause (CSVD vs. other pathologies). In fact, the prevalence of MBs in QSM in healthy participants of this small cohort is higher than the reported prevalence of sporadic CSVD in pathological studies of community-dwelling cohorts (~23% for CAA and ~35% for arteriolosclerosis) (46, 47). Nonetheless, it must be reminded that the majority of these studies relied on histological examination of a small portion of the brain tissue for this diagnosis, thus possibly underestimating the prevalence of CSVD pathology.

The more precise assessment of MBs thanks to the use of QSM could also have implications to better estimate the risk of brain hemorrhage, the deadliest clinical event related to CSVD, which has been positively associated to the number of MBs (7, 8) and leads to concrete clinical decisions, such as the use and selection of an anticoagulant. A more precise monitoring of MBs could also become necessary within the context of the recently approved drug against AD, Aducanumab (48) which demonstrated increased incidence of MBs and cSS in treated patients (49), with clinical consequences that remain to be estimated. Our observations and those of many similar studies, are based on expensive and not widely available methods. The increasing meaning attributed to the interaction between CSVD and neurodegenerative disease calls for studies, that aim to validate and then introduce more convenient biomarkers (e.g. serological).

Besides the detection of MBs, further QSM-applications could be meaningful for CSVD (50). For example, overall iron overload could be detected in the tissue, as it has been in some neurodegenerative diseases (51, 52). Furthermore, the identification of chronic inflammatory activity (53, 54), blood-brain-barrier leakage (55), and diffuse demyelination (56) could give better insight into the pathophysiology of CSVD. Due to these possibilities, the last years have seen the surge of a number of open access toolboxes for QSM reconstruction, which will increasingly facilitate the implementation of QSM for clinical purposes (57). The use of multi-echo data (58) and of techniques to achieve susceptibility source separation (56), would have allowed a more precise assessment of the susceptibility within the brain tissue. However, because our aim was to detect round/ovoid structures, such as MBs and MBs-mimics, these factors would have likely not significantly impacted the results of the study. The main limitation of our study is that a comparison between the number of MBs observed with QSM and those observed with the widely used SWI is missing. Future projects which rely on such data sets should answer this question. One further limitation of our study is the relatively low number of participants. However, the size of our cohort is comparable to that of previous 7T MRI studies (59) and must be set in relationship to the many contraindications that apply to high field imaging. The same contraindications could have also led to a selection bias toward healthier participants.

The present study is the first to systematically report the improved precision in MBs detection at high-field and high-resolution QSM in healthy elderly human participants and patients with CSVD. Using this post-processing method could have implications for the classification of CSVD patients and could more accurately mirror the presence of cerebral small vessel pathology. Broader studies are needed to confirm these results.

## Data availability statement

The raw data supporting the conclusions of this article will be made available by the authors, without undue reservation.

## Ethics statement

The studies involving human participants were reviewed and approved by University Clinic of Magdeburg. The patients/participants provided their written informed consent to participate in this study.

## Author contributions

VP collected the data, designed the study and performed the analysis, and drafted the manuscript together with JR. SS mentored the study. RY and FS helped with the QSM reconstruction. HK contributed to the analysis of the microbleeds. All authors revised and corrected the manuscript. All authors contributed to the article and approved the submitted version.
